# ﻿A phylogenetic study of *Micareamelaeniza* and similar-looking species (Pilocarpaceae) unveils hidden diversity and clarifies species boundaries and reproduction modes

**DOI:** 10.3897/mycokeys.106.123484

**Published:** 2024-07-05

**Authors:** Annina Kantelinen, Måns Svensson, Jiří Malíček, Jan Vondrák, Göran Thor, Zdeněk Palice, Stanislav Svoboda, Leena Myllys

**Affiliations:** 1 Botany and Mycology Unit, Finnish Museum of Natural History, University of Helsinki, P.O. Box 7, FI-00014 Helsinki, Finland University of Helsinki Helsinki Finland; 2 Museum of Evolution, Uppsala University, Norbyvägen 16, SE-752 36 Uppsala, Sweden Uppsala University Uppsala Sweden; 3 Czech Academy of Sciences, Institute of Botany, Zámek 1 252 43, Průhonice, Czech Republic Czech Academy of Sciences, Institute of Botany Průhonice Czech Republic; 4 Department of Botany, Faculty of Science, University of South Bohemia, CZ-37005 České Budějovice, Czech Republic University of South Bohemia České Budějovice Czech Republic; 5 Department of Ecology, Swedish University of Agricultural Sciences, P.O. Box 7044, SE-750 07 Uppsala, Sweden Swedish University of Agricultural Sciences Uppsala Sweden

**Keywords:** Biodiversity, DNA-barcoding, lichenized ascomycete, new species, overlooked taxa, reproduction mode

## Abstract

*Micarea* (Ascomycota, Pilocarpaceae) is a large cosmopolitan genus of crustose lichens. We investigated molecular systematics and taxonomy of the poorly known *Micareamelaeniza* group focussing on *M.melaeniza*, *M.nigella* and *M.osloensis*. A total of 54 new sequences were generated and using Bayesian and maximum likelihood analysis of two markers (nuITS and mtSSU), we discovered two previously unrecognized phylogenetic lineages, one of which is described here as *Micareaeurasiatica* Kantelinen & G. Thor, **sp. nov.**, morphologically characterized by pycnidia that are sessile to emergent, cylindrically shaped, with greenish-black K+ olive green, wall pigmentation and containing large mesoconidia up to 6 µm in length. The species is known from Japan and Finland. In addition, we show that the reproduction biology of *M.osloensis* has been poorly understood and that the species often occurs as an anamorph with stipitate pycnidia. We present a species synopsis and notes on pigments. Our research supports previous results of asexuality being an important reproductive strategy of species growing on dead wood.

## ﻿Introduction

**Figure 1.**
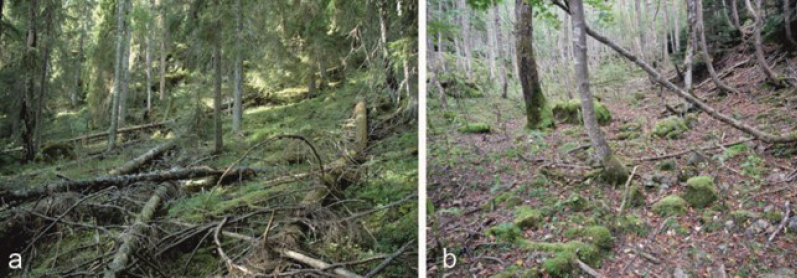
Typical habitats for species in the *M.melaeniza* group in boreal and boreonemoral forests **a** Koli National Park, Eastern Finland (photo: Kantelinen) **b** Nikko National Park, Central Honshu, Japan near the type locality of *M.eurasiatica* sp. nov. (photo: Thor).

Species of the genus *Micarea* Fr. are lichenized ascomycetes belonging to the family Pilocarpaceae. Currently, more than 140 species are known, and new species are continually described (e.g., Czarnota 2007; Czarnota and Guzow-Krzemińska 2010; Sérusiaux et al. 2010; Guzow-Krzemińska et al. 2016, 2019; van den Boom et al. 2017; Kantvilas and Coppins 2019; Launis and Myllys 2019; Launis et al. 2019a, b; van den Boom et al. 2020; Vondrák et al. 2022; Index Fungorum 2023). *Micarea* species are globally distributed with representatives found on all continents. These species occur across a wide range of habitats and grow on substrates such as bark, dead wood, rocks, soil and bryophytes. Some *Micarea* species are specialized and prefer specific habitats, such as dead wood in old-growth forests (Fig. 1). Their typical substrate has acidic pH (e.g. Coppins 1983; Czarnota 2007).

Despite the diversity and global presence of *Micarea* species, they are often overlooked and poorly understood. Several factors contribute to the challenge of identifying them. First, they are typically small. Second, *Micarea* exhibits a wide range of sexual and asexual propagules, including ascospores, three types of conidia (micro-, meso-, and macroconidia), and thallus fragments called goniocysts which likely serve as asexual propagules including both symbiotic partners. Some *Micarea* species are primarily sexual, while others often lack sexual structures but form numerous pycnidia where asexual conidia are produced (e.g. Coppins 1983; Czarnota 2007; Kantelinen et al. 2022a). Third, some *Micarea* species display intraspecific colour variations, which depend on light exposure and corresponding pigment levels. For example, the Sedifolia-grey pigment, commonly found in the apothecia of *Micarea* species, varies in concentration from light grey in shaded situations to almost black in well-lit habitats (Coppins 1983; Czarnota 2007; Launis et al. 2019a, 2019b). Due to these challenges, identifying *Micarea* species typically requires careful examinations of microscopic features, chemical testing (spot tests and TLC) and/or DNA sequencing.

Significant progress in the understanding of species boundaries and diversity within *Micarea* has been achieved through the application of molecular methods (Czarnota and Guzow-Krzemińska 2010; Sérusiaux et al 2010; Guzow-Krzemińska et al. 2016; van den Boom et al. 2017; Launis et al. 2019a, b). However, some infra-generic groups in *Micarea* have been more studied than others. One group that remains poorly understood and has been sequenced only rarely is *Micareamelaeniza* Hedl. and similar species, i.e. *M.anterior* (Nyl.) Hedl., *M.botryoides* (Nyl.) Coppins, *M.deminuta* Coppins, *M.denigrata* (Fr.) Hedl., *M.melaeniza*, *M.misella* (Nyl.) Hedl., *M.nigella* Coppins, *M.olivacea* Coppins and *M.osloensis* (Th. Fr.) Hedl.. These species share morphological characteristics, such as a thin or endosubstratal thallus, small (0.1–0.3 mm wide) dark apothecia and/or dark stipitate mesopycnidia, as well as often simple to one septate ascospores. Furthermore, *M.botryoides*, *M.deminuta*, *M.melaeniza*, *M.nigella*, *M.olivacea* and *M.osloensis* are similar in having dark hypothecium and dimorphic paraphyses. The Cinereorufa-green (K+ green, HNO_3_+ purple) and Superba-brown (K–, HNO_3_–) pigments are often present, as well as sometimes Melaena-red (K+ green, HNO_3_+ purple-red). Most of these species are obligate or facultative lignicoles (Coppins 1983, Czarnota 2007). Despite their morphological and ecological similarities, all the species are not necessarily closely related (Coppins 1983; Andersen and Ekman 2005; Sérusiaux et al. 2010).

We aim to clarify the molecular systematics and morphology of the poorly known *M.melaeniza* and its similar-looking species, focusing especially on *M.melaeniza*, *M.nigella* and *M.osloensis*, species that are morphologically challenging to identify. We use phenotypic characters and sequence data from two loci (nuITS and mtSSU). Additionally, we also address the species ´ reproduction biology. Our study generates reliable sequences of several rarely collected species and furthers understanding on lichen diversity in boreal, boreonemoral and hemiboreal forests especially on dead wood and conifer bark.

## ﻿Materials and methods

A substantial portion of the sequenced specimens in this study were collected from southern and central Finland as part of a research project that investigated lichen diversity on dead wood (years 2012–2014, Kantelinen et al. 2022b). Logs and stumps of decaying *Piceaabies* trees in decay stages 2, 3, 4 and 5 were inventoried (following Renvall 1995).

In addition, specimens were obtained from the Czech Republic, Japan, Russian Caucasus, Sweden and Ukraine. These specimens were collected from dead wood and bark and sequenced when possible. Geo-coordinates are given in the format WGS84. Relevant specimens were also looked for amongst fresh *Micarea* collections from Australia, Brazil, Kenya, Rwanda and Tasmania with no success. Herbarium collections and type specimens from the herbaria FR, H, PRA, RBGE, S and UPS were studied.

### ﻿Species identification

Specimens were identified with a dissecting (Leica S4E) and compound (Leica DM750) microscopes. Anatomical characters and ascospore dimensions were measured in water and K. The number of measured ascospores and conidia depended on their availability, but usually 10–30 were measured and the rest were examined superficially to ensure that they fell into the same size category. To detect and determine the insoluble pigments present in the specimens chemical spot tests with 10% potassium hydroxide (K) and sodium hypochlorite (C) and nitric acid (HNO_3_) were used (Orange et al. 2010). Fresh material was often not sufficient for thin-layer chromatography (TLC). The specimens are generally quite small and have no or thin thallus, meaning that a substantial part of the collection would need to be taken for TLC. No secondary substances have previously been detected in *M.melaeniza*, *M.nigella* or *M.osloensis*, nor in the similar-looking species *M.anterior*, *M.misella* and *M.substipitata* Palice & Vondrák (Coppins 1983; Czarnota 2007; Vondrák et al. 2022). Thus, TLC is of limited practical value in the study of this species group.

Total genomic DNA was extracted from lichen structures (apothecia, pycnidia and/or thallus) in labs in Finland and the Czech Republic. In Finland, extractions were conducted using DNeasy® Blood & Tissue kit by Qiagen following the manufacturer´s instructions with the following exceptions. Lichen structures of approximately 0.5–1 mm in diam. were ground with mini-pestles in 40 µl of lysis buffer, after which 140 µl of the buffer was added by simultaneously flushing lichen fragments from the mini-pestle. The extracted DNA was eluted in 50 µl of the eluation buffer. Samples were incubated for 5 minutes and centrifuged. After the first elution, a second elution was performed to increase sample availability by adding another 50 µl of the elution buffer, incubated for 5 minutes and centrifuged. The two elutions were stored in the freezer in separate microcentrifuge tubes. In the Czech Republic, extractions were conducted using ISOLATE II DNA Plant Kit (Bioline) according to the manufacturer´s protocol using a cetyltrimethylammonium bromide (CTAB)-based protocol (Aras and Cansaran 2006).

In Finland, PCR reactions were prepared using PuReTaq Ready-To-Go PCR beads (GE Healthcare). The 25 µl reaction volume contained 19 µl dH2O, 0.4 µM of each primer and 4 µL extracted DNA. The ITS and mtSSU regions were used for species identification. PCR was run under the following conditions: initial denaturation for 10 min at 95 °C followed by six cycles of 1 min at 95 °C (denaturation), 1 min at 62 °C (annealing), and 1 min 45 s at 72 °C (extension); for the remaining 35 cycles, the annealing temperature was decreased to 56 °C; the PCR program ended with a final extension of 10 min at 72 °C. The primers used were ITS1LM and ITS2KL (Myllys et al. 1999), and mrSSU1 and mrSSU3R (Zoller et al. 1999) and they were used for PCR amplification and sequencing. In the Czech Republic, PCR reactions for Malíček´s specimens were prepared following the protocol in Malíček et al. (2023). For Vondrák´s and Palice´s specimens PCR reactions were prepared as follows: Polymerase chain reactions were performed in a reaction mixture containing 2.5 mmol/l MgCl2, 0.2 mmol/l of each dNTP, 0.3 µmol/l of each primer, 0.5 U Tag polymerase (TOP-Bio, Praha, Czech Republic) in the manufacturer’s reaction buffer, and sterile water to make up a final volume of 10 µl. The ITS and mtSSU regions were used for species identification. PCR was run under the following conditions: for ITS initial denaturation for 3 min at 94 °C followed by 30 cycles of 30 sec at 94 °C (denaturation), 30 sec at 56 °C (annealing), and 2 min at 72 °C (extension); the PCR program ended with a final extension of 7 min at 72 °C. For mtSSU initial denaturation for 10 min at 94 °C followed by 40 cycles of 30 sec at 94 °C (denaturation), 30 sec at 58 °C (annealing), and 2 min at 72 °C (extension); the PCR program ended with a final extension of 7 min at 72 °C. The primers used were ITS1F (White et al. 1990) and ITS4 (Gardes and Bruns 1993), and mrSSU1 (Zoller et al. 1999) and mrSSU7 (Zhou and Stanosz 2001), and they were used for PCR amplification and sequencing.

### ﻿Phylogenetic analyses

To examine the phylogenetic position of our study species within *Micarea* s. lat., we ran a preliminary analysis of an mtSSU data matrix using *Psoradecipiens* (Hedw.) Hoffm. from the family Psoraceae as an outgroup, based on the studies by Andersen and Ekman (2005) and Sérusiaux et al. (2010). In the phylogeny (tree not shown) our new samples fall outside of *Micarea* s. str. (i.e. the *M.prasina* group) and close to *M.doliiformis* (Coppins & P. James) Coppins & Sérus., *M.paratropa* (Nyl.) Alstrup, *M.assimilata* (Nyl.) Coppins and *Leimoniserratica* (Körb.) R.C. Harris & Lendemer (see phylogeny in Sérusiaux et al. 2010).

The final phylogenies, including 29 newly generated mtSSU and 25 ITS sequences (Table 1), were first aligned with MUSCLE v.3.8.31 (Edgar 2004) using the European Molecular Biology Laboratory, European Bioinformatics Institute’s (EMBL-EBI) freely available web server (http://www.ebi.ac.uk/Tools/msa/muscle/). Based on our previous studies (Launis et al. 2019a, b) and our preliminary phylogenetic reconstruction of the genus, *M.byssacea* and *M.prasina* belonging to *Micarea* s. str. were selected as outgroups. We ran separate single-marker analyses by using MrBayes3.2.7a and did not detect conflicting clades between the analyses, although missing data was higher in the ITS matrix, and hence decided to concatenate the data by using Mesquite v. 3.61 (Maddison and Maddison 2023). The two-locus data matrix from sequences of 43 specimens included 1245 aligned nucleotide characters, with 707 positions in the mtSSU and 537 positions in the ITS regions. The hypervariable region at the end of the mtSSU was removed from the analyses. *Micareadoliiformis* is represented by sequences from two specimens that were combined as one, the other specimen represented by an mtSSU sequence and another by ITS sequence (see Table 1). The data matrix was subjected to Bayesian inference using MrBayes (v. 3.2.7a) (Ronquist and Huelsenbeck 2003) and to maximum likelihood (ML) analysis using freely available IQ-Tree 1.6.12 (Trifinopoulos et al. 2016) web server (http://iqtree.cibiv.univie.ac.at/). For the Bayesian analysis, substitution models were selected by having the MCMC procedure sample across models (Huelsenbeck et al. 2004). The convergence of the four parallel runs was checked after 2000000 generations using Tracer (v. 1.5) (Rambaut et al. 2018) and graphed using FigTree (v. 1.4.4). For the ML analysis, model TIM2+F+I+G4 was chosen by having IQ-Tree run the best-fitting substitution model for our one partition matrix, and branch lengths were assumed to be proportional across subsets. Node support was estimated with 1000 bootstrap replicates using the ultrafast bootstrap algorithm. The alignment is available from the Dryad Digital Repository https://doi.org/10.5061/dryad.79cnp5j44.

**Table 1.** List of *Micarea* specimens used in the phylogenetic analysis with locality, voucher information and GenBank accession numbers.

Taxon	Locality	Voucher information, sequence ID	mtSSU	ITS
*M.anterior*	Finland	Kantelinen 199 (H), A265	PP811702	PP811675
*M.botryoides*	Norway	Andersen 79b (BG)	AY567741	AY756471
*M.byssacea*	Finland	Kantelinen (Launis) 289103 (H), A98	MG707768	MG521562
*M.contexta*	Finland	Kantelinen 1914 (H), A569	PP811722	PP811692
*M.deminuta*	Japan	Thor 40245 (UPS), A926	PP811719	PP811689
*M.deminuta*	Czech Republic	Palice 6745 & Voříšková (PRA)	AY756446	AY756474
*M.denigrata*	Finland	Kantelinen 723 (H), A686	PP811721	PP811691
*M.doliiformis*	UK, Wales	Orange, LG database 29 (LG)	GU138666	
*M.doliiformis*	UK	Andersen 178a (BG)		HQ650654
*M.eurasiatica* sp. nov.	Finland	*Kantelinen* 2729 (H), A466	PP811712	PP811684
*M.eurasiatica* sp. nov.	Japan	*Thor* 40053 (UPS) A914, holotype	PP811720	PP811690
*M.eximia*	Finland	Kantelinen 3785 (H), A785	MT982134	MT981600
*M.globulosella*	Finland	Kantelinen (Launis) 67114 (H), A243	MG707744	MG521547
*M.incrassata*	Finland	*Kantelinen* 90 (H), A90	MT982132	MT981598
*Micarea* sp.	Finland	*Kantelinen* 2640 (H), A487	PP811703	PP811676
*M.melaeniza*	Finland	*Kantelinen* 2430 (H), A772	PP811709	PP811682
*M.melaeniza*	Ukraine	*Vondrák* 21921 (PRA)	PP811724	PP811694
*M.melaeniza*	Russia	*Vondrák* 23358 (PRA)	PP811725	PP811695
*M.melaeniza* (*M.nigella* in GenBank)	Czech Republic	*Palice* 32013 (PRA)	OQ646318	
*M.misella*	Finland	*Kantelinen* (Launis) 108111 (H), A264	MG707742	MG521545
*M.nigella*	Finland	*Kantelinen* 1971 (H), A589	PP811706	PP811679
*M.nigella*	Finland	*Kantelinen* 1974 (H), A588	PP811714	PP811686
*M.nigella*	Finland	*Kantelinen* 1921 (H), A572	PP811715	PP811687
*M.nigella*	Czech Republic	*Malíček* 16287	PP811726	PP811696
*M.nigella*	Czech Republic	*Malíček* 14664	PP811729	PP811698
*M.osloensis*	Finland	*Kantelinen* 1685 (H), A574	PP811704	PP811677
*M.osloensis*	Finland	*Kantelinen* 1865 (H), A575	PP811705	PP811678
*M.osloensis*	Finland	*Kantelinen* 1909 (H), A583	PP811717	
*M.osloensis*	Finland	*Kantelinen* 2899 (H), A736	PP811716	PP811688
*M.osloensis*	Finland	*Kantelinen* 1923 (H), A573	PP811713	PP811685
*M.osloensis*	Finland	*Kantelinen* 1686 (H), A768	PP811708	PP811681
*M.osloensis*	Finland	*Kantelinen* 2643 (H), A484	PP811710	PP811683
*M.osloensis*	Finland	*Kantelinen* 2003 (H), A594	PP811707	PP811680
*M.osloensis*	Finland	*Kantelinen* 2648 (H), A485		PP811693
*M.osloensis*	Czech Republic	*Malíček* 16281	PP811730	
*M.osloensis* (*M.melaeniza* in GenBank)	Czech Republic	*Palice* 30267 (PRA)	OQ646316	
*M.osloensis* (*M.nigella* in GenBank)	Czech Republic	*Palice* 30266 (PRA)	OQ646320	OQ717948
*M.osloensis*	Sweden	*Svensson* 4385 (UPS), C792	PP811718	
*M.osloensis*	Russia	*Vondrák 22836* (*PRA*)	PP811723	
*M.osloensis* (*M.melaeniza* in GenBank)	Czech Republic	*Vondrak* 25774 (PRA)	OQ646315	OQ717944
*M.osloensis*	Czech Republic	*Vondrák* 19007 (PRA)	PP811727	PP811697
*M.osloensis*	Czech Republic	*Vondrák* 22217 (PRA)	PP811728	
*M.prasina*	Finland	*Kantelinen (Launis)* 265101 (H), A92	MG707747	MG521549
*M.substipitata*	Finland	*Kantelinen* 2700 (H), A469	PP811711	

## ﻿Results

**Figure 2.**
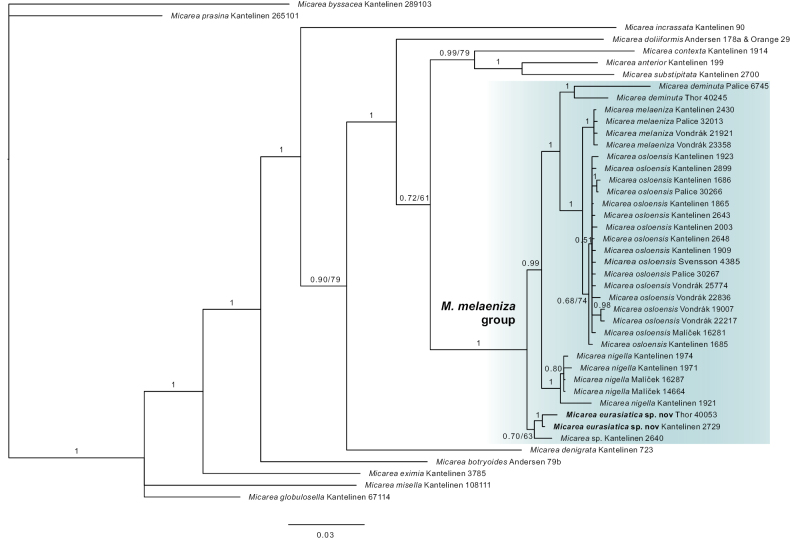
Bayesian tree of the *Micareamelaeniza* -group and similar-looking species based on mtSSU and ITS sequences. Bayesian posterior probabilities are always indicated near the branches together with bootstrap support when less than 80 (e.g. 0.70/63).

We present the first molecular phylogeny of *M.melaeniza* and similar-looking species. We recorded 72 specimens of the *M.melaeniza* group of which 26 were sequenced successfully. In addition, we downloaded sequences from GenBank (Table 1). The topologies of the Bayesian and ML analyses showed no conflict and hence only the Bayesian tree is shown (Fig. 2).

The phylogenies are well-supported and resolved into 18 taxa. *Micareamisella*, *M.globulosella*, *M.eximia* and *M.botryoides* form basal nodes for the phylogeny. The remaining taxa resolve into four clades that include the *M.melaeniza* group and its sister groups. The *M.melaeniza* group, delimited in this study, includes six monophyletic taxa, i.e. *M.deminuta*, *M.melaeniza*, *M.nigella*, *M.osloensis* and two undescribed species, each represented by two specimens. We describe the first as *M.eurasiatica* Kantelinen & G. Thor, sp. nov., but refrain from describing the other because of insufficient material and sequence data (“Kantelinen 2640 and Kantelinen 1870”; sequences from the latter specimen are not of sufficient quality and therefore not included in the final analyses even though it formed a monophyletic clade in preliminary analysis). Based on our results the similar looking *M.anterior*, *M.contexta* and *M.subtsipitata* are not included in the *M.melaeniza* group. Instead, they form a sister clade to this group, but the relationship receives no support and is based on limited taxon sampling. Several outgroups, including *M.incrassata*, *M.doliiformis*, and *Lecaniacyrtella* have been tested but the overall topology of the phylogeny has not changed. The main distinguishing morphological characters are presented in the species synopsis Table 2. Pigments are presented in Table 3.

**Table 2.** Species synopsis representing key morphological characters based on our study and literature (Hedlund 1892; Coppins 1983; Czarnota 2007).

	reproduction	anatomical coloration	spores (simple, if not mentioned otherwise)	paraphyses (μm wide)	mesopycnidia	mesoconidia	K-reaction
*Micareaanterior*	pycnidia, ap quite rare	(orange-/reddish-)brown	8–12(–12.5) × 3–4(–4.8) μm 0–1(–2) sept.	one type: 1–1.5 μm, sometimes upper part 2.5–3 μm	stipitate, brown from base and black from the top, up to 250 μm tall (Finnish specimens); sessile to stipitate, pallid, often with reddish-brown blotches, up to 250 μm tall (Coppins 1983); dilute brownish (Czarnota 2007)	3.5–4.5 × 1.2–1.5 μm	no reaction or dulling
*M.contexta*	apothecia, pycnidia rare	dark green, purple	7–13(–14) × 3–4.6 μm	two types: : thick 1.5–2.0 μm; thin less than 1 μm	sessile to emergent, c. 40 μm diam.	4.2–5.0 × 1.2–1.5 μm	K+ green
*M.deminuta* (TYPE)	apothecia	dark-brown epihymenium, brown-black (not warm) [dark ± reddish brown / Coppins 1995] hypothecium	7–11(–11.8) × 4–5(–5.5) μm; (5.8–)7.7–10.1(–11.5) × (3.2–)3.7–4.5(–4.8) μm (Coppins 1995)	two types: thick 1.5–2.0(–3.0) μm; thin 0.8–1.0 μm	no emergent/stalked pycnidia	not reported	K+ green
*M.deminuta* (sensu Czarnota 2007)	apothecia, pycnidia rare	olivaceous epihymenium (with dull brown to brown-black streaks in hymenium), brown-black hypothecium	(7.2–)8.2–11.3(–11.8) × (4.1–)4.4–5.5(–6.1) μm	two types: thick (1-)1.2–2.0 μm; thin 0.8–1.0 μm	emergent, often with gaping ostioles, black-brown with brown to olive-brown walls	(4.9–)5.5–8(–8.1) × 1.5–1.8 um	K- or K+ dull olive grey [=K+ green]
*M.misella*	pycnidia, ap not always present	dark olive-brown epihymenium, hyaline hymenium and hypothecium	6–9(–10) × (2–)2.5–3.5(–4) μm; 0(–1) sept.	one type: (0.8–)1–1.2(–1.5) μm	brown to black, glossy, stipitate up to 300 μm tall, walls olive, olive-brown	3.5–5(–6.5) × 1.2–1.4(–1.7) μm	K+ violet
*M.melaeniza* (TYPE)	ap and pycnidia	brownish-black, greenish	5–9.0 × 2.5–3.8 μm	two types: thick c. 2 μm; thinner c. 0.7–1 μm	black, stipitate up to 300 μm tall, upper part of the walls greenish-brown and lower part reddish-brown sometimes growing from the same base	3–3.5 × 1.75 μm	K+ green, or rarely K–
*M.melaeniza* (sequenced)	pycnidia, ap rare	brownish-black, greenish, rarely purplish tone	5–9.0 × 2.5–3.8 μm	two types: thick c. 2 μm; thinner c. 0.7–1 μm	black, stipitate up to 300 μm tall, upper part of the walls greenish-brown and lower part reddish-brown, rarely purplish tone, sometimes growing from the same base	2.5–3.5 × 1.2–1.8 µm	K+ green, or rarely K–
*M.nigella* (TYPE)	ap and pycnidia	brownish-black, often purple tinge	6.5–12 × 2.5–4 μm	two types: thick 2–3 μm; thinner c. 0.7–1 μm	black, stipitate, purplish-brown according to the description, but the type specimen is not clearly purplish, but walls are instead brown-black to slightly greenish-black	3.4–4.3 (–4.5) × 1.2–1.6 (–1.8) μm	K+ green
*M.nigella* (sequenced)	pycnidia, no ap	brownish-black, often purple tinge	none	none	black, stipitate, walls brownish black or often purplish black, sometimes growing from the same base	(3.5–)3.8–4.5 × 1.2–1.5(–1.8) μm	K+ green
*M.olivacea* (TYPE)	ap and pycnidia	olivaceous, olive-brown	(7–)9–12.3 × 2.5–3.5 μm	two types: thick 2–3 μm; thinner c. 1–1.2 μm	sordid green, numerous, inconspicuous, +/-immersed	3.4–4.3 × 1.2–1.6 μm	K+ green
*M.osloensis* (TYPE)	ap, no pycnidia	warm brown tones	6–9.5(–10) × 3–4 μm	two types: mostly 2–3 μm, unevenly shaped, sometimes branched; thinner 1.5–1.8 μm	not seen	not seen	No reaction
*M.osloensis* (sequenced)	pycnidia, ap few	warm brown and olivaceous tones	7–10 × 3.0–3.5 (–4.0) μm	two types: thick 2–3 μm often uneven in shape; thinner c. 1–1.5 μm	often numerous and crowded, simple or branched from the base, emergent or shortly stalked up to 180 μm, dark brown to blackish, walls greenish black to greenish brown from the top with a warm brown lower part	3.5–4.5(–5) × 1.2–1.5(–1.8) μm	No reaction, or K+ green epihymenium
*M.eurasiatica* sp. nov.	pycnidia, ap few	greenish black, dark brown	(6–)7–9 × 3–4 µm	two types: thick 2–3 μm sometimes branched; thinner c. 0.7–1.5 µm	sessile to emergent, 50–80 (–100) μm tall, 30–45 μm wide	4.5–6.0 × 1.2–1.5(–1.8) μm	K+ green
*M.* sp. (Kantelinen 2640)	pycnidia, ap few	brownish-black, greenish-black, violet-black	none seen	two types	sessile (to emergent), c. 50 μm tall, 50 μm wide	4.2–5.0 × 1.2–1.5 μm	K+ green
*M.substipitata*	pycnidia, ap not always present	pallid to whitish, hyaline	7–10 (–11) × 2.2–3.5 (–3.8) μm (0–) 1 sept.	two types: mostly 0.9–1.3 μm; rarely thicker 1.5–2.0 μm	sessile to shortly stipitate, white, up to 250 μm tall	2.5–3.5(–4) × 1.0–1.5 μm	No reaction

**Table 3.** Pigments found in apothecia and pycnidia based on our study and literature (Hedlund 1892; Coppins 1983; Czarnota 2007).

	Cinereorufa-green [Pigment A] *K*+ green intensifying, *HNO_3_*+ purple	Melaena-red [Pigment B] *K*+ dull sordid green, *HNO_3_*+ purple-red	Melaenida-red [Pigment C] *K*+ purplish-red, *HNO_3_* -	Superba-brown [within Pigment F] No reaction in *K* or *HNO_3_*
*M.deminuta*	x			x
*M.melaeniza*	x	(x)	(x)	x
*M.nigella*	x	x	x	
*M.osloensis* (type)				x
*M.osloensis* (new)	(x)			x
*M.eurasiatica* sp. nov	x		(x)	x

In addition to our specimens, we downloaded *M.melaeniza* and *M.nigella* sequences from Genbank. Based on our phylogenetic analyses, all of them fall inside *Micareaosloensis* (OQ646318, OQ646320, OQ717948, OQ646315, OQ717944, OQ646316, in addition OQ717947 and OQ646319 are identical with others and excluded from our final analysis because of repetition). Three sequences from Genbank were left out from our analyses (AY756488, AY756484, OQ717944), because they are substantially different compared to the other sequences in the *M.melaeniza* group and hence the alignment and phylogenetic analysis became unreliable. Based on blast searches AY756484 is a species of *Lepraria*, AY756488 perhaps *Micareamelaena* or *M.nitschkeana*, and OQ717944 an uncultured fungus.

Our data include a taxon with a high morphological resemblance to *Micareaosloensis*, a species found only twice before in years 1874 and 2007. Unfortunately, we were not successful in sequencing the old collections. Despite the high morphological resemblance, our new specimens have some subtle differences compared to the type. The type specimen of *M.osloensis* is a fertile specimen with apothecia, whereas our specimens are usually asexual with pycnidia. The specimens are mostly dimorphic, meaning that the existing sequenced specimens are usually either sexual or asexual, and rarely both. In addition, some of the fresh material is with Cinereorufa green pigment which is not present in the type. See taxonomy section for further info.

### ﻿Taxonomy

#### ﻿ Micarea eurasiatica

Taxon classificationFungiLecanoralesPilocarpaceae

Kantelinen & G. Thor sp. nov.

29C08607-4D49-5D6C-BF00-A2F7713E4C9A

MycoBank No: 854181

Fig. 3A, B

##### Type.

Japan, Honshu, Gunma Prefecture, Katashina-mura, Nikko National Park, 4.7 km E of Marunuma Kogen Ski Resort, 550 m S of the parking lot at the start of the trail up to the summit of Mt. Oku-Shirane, along the trail. Open forest with mainly deciduous trees. On *Tsugadiversifolia* log. 36.81573°N, 139.37823°E (± 10 m), elevation 1791 m. 2019. Thor 40053 (Holotype UPS). DNA sample A914.

**Figure 3.**
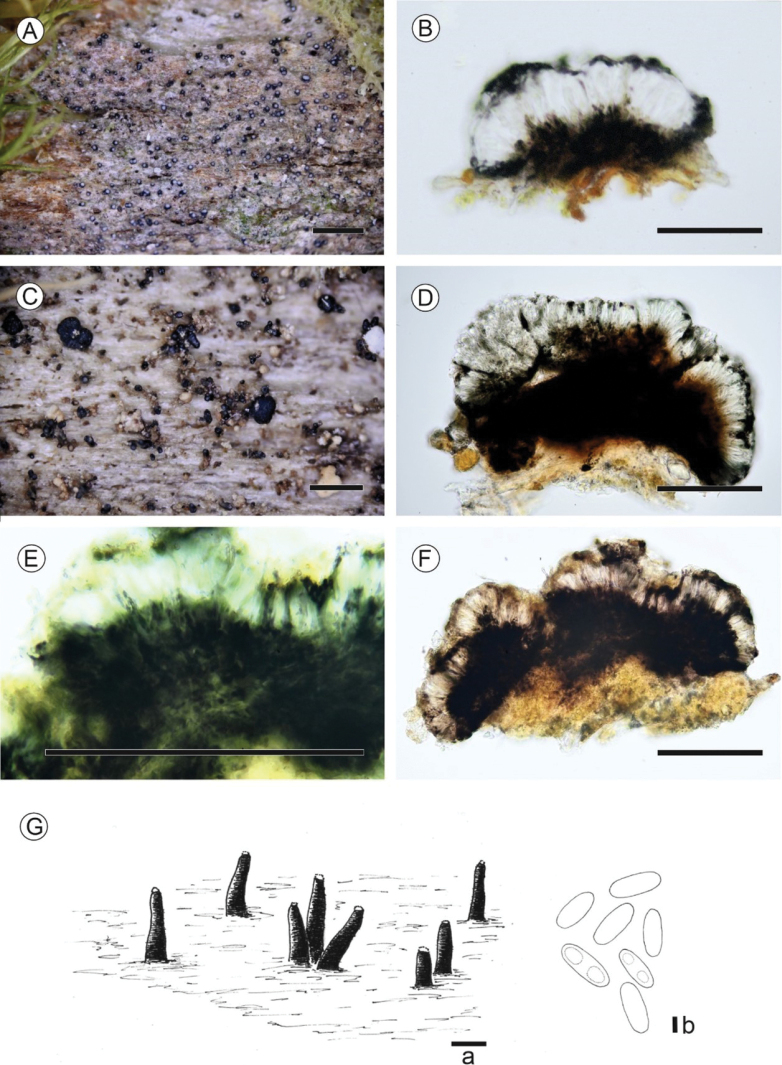
Morphological and anatomical features **A, B***Micareaeurasiatica* sp. nov. (Thor 40053) **A** habit **B** apothecial section in water **C, D***Micareamelaeniza* (Holotype) **C** habit, apothecia and pycnidia **D** apothecial section in water **E–G***Micareanigella* (Holotype) **E** apothecial section in K **F** apothecial section in water **G** drawing of *M.nigella* pycnidia on dead wood (Kantelinen 1974, H), Ga.) Mesopycnidia extruding mesoconidia, Gb.) Mesoconidia are cylindrical, ellipsoid, sometimes biguttulate. Photos and drawing Kantelinen. Scale bars: Habit 0.5 mm (**A, C**); Apothecial sections 100 µm (**B, C, E, F**); Drawing Ga 100 µm, Gb 1 µm.

##### Description.

***Thallus*** endoxylic. Photobiont micareoid, 4–7 µm.

***Apothecia*** few, immarginate, convex, black, matt, 0.1–0.2 mm in diam. Hymenium 25–40 µm tall, hyaline or tinged green, K+ greenish when tinged, sometimes with darker vertical streaks. Epihymenium black to blackish-green, K+ green, HNO_3_+ purple (Cinereorufa-green). Asci clavate, 28–38 × 11–14 µm. Ascospores ellipsoid to ovoid, simple, (6–)7–9 × 3–4 µm. Paraphyses numerous, of two types: 1. evenly distributed, branched, thin, c. 0.7–1.5 µm wide, 2. evenly distributed, stout, sometimes branched, 2–3 µm wide, not always coated in pigment. Hypothecium c. 35–45 µm tall, dark brown, K – (Superba-brown), or sometimes with a slight purple tinge (Melaenida-red), hyphae coated with dark brown pigment. Excipulum not evident.

***Mesopycnidia*** few to abundant, sessile to emergent, cylindrical in shape, 50–80(–100) µm tall, 30–45 µm wide, black, walls greenish black, K+ green, HNO_3_+ purple (Cinereorufa-green), sometimes merged from base, usually extruding white mass of conidia that sometimes merge with neighbouring conidial mass. Mesoconidia ellipsoid-cylindrical, (4–)4.5–6.0 × 1.2–1.5(–1.8) µm. Micro- or macropycnidia not seen.

***Chemistry*.** Material not sufficient for TLC.

***Crystalline granules*** not present in apothecia or pycnidia.

##### Habitat and distribution.

*M.eurasiatica* is currently known from Finland and Japan. In Finland, the species was collected in a shaded and dense, *Piceaabies* dominated managed forest. In Japan, the species occurred in a semiopen forest with mainly deciduous trees. On both occasions, the species was found growing on dead wood.

##### Notes.

*M.eurasiatica* is currently known from two collections. The type collection has abundant mesopycnidia, and additionally few small apothecia. The other collection has only pycnidia. The most important diagnostic characters are the combination of sessile to emergent pycnidia that are cylindrical in shape, greenish-black pycnidial walls, large mesoconidia (up to 6 µm in length) and a K+ olive green reaction (Cinereorufa-green). If apothecia are present, they are 0.1–0.2 mm wide, have greenish-black epihymenium (K+ green, Cinereorufa-green) and dark brown hypothecium (K– or sometimes slightly K+ purple if Melaenida-red present). *Micareaeurasiatica* resembles other often asexual *Micarea* species on dead wood such as *M.melaeniza*, *M.misella*, *M.olivacea* and *M.osloensis*. *Micareaeurasiatica* differs from *M.melaeniza* by having shorter mesopycnidia, longer mesoconidia (*M.melaeniza*: (3–)3.5–4.5 × 1.2–1.8 µm, *M.eurasiatica*: (4–)4.5–6.0 × 1.2–1.5(–1.8) µm), and a more greenish black wall colouration in the pycnidia with no brown tones. *Micareamisella* has a K+ violet reaction (Sedifolia-grey) instead of K+ olive green and its mesopycnidia are brownish-black and taller (Coppins 1983; Czarnota 2007). *Micareaolivacea* has rather similar short mesopycnidia that react K+ green, but the wall of pycnidia is olive-brown and the mesoconidia are shorter than those of *M.eurasiatica* (*M.olivacea*: 3.4–4.3 × 1.2–1.6 µm, *M.eurasiatica*: (4–)4.5–6.0 × 1.2–1.5(–1.8) µm) (Coppins 1983). *Micareaosloensis*, on the other hand, has a warm brown wall colouration and usually no K reaction.

##### Additional specimen studied.

**Finland, Uusimaa**, Tuusula, W of Korso, shaded and dense *Piceaabies* dominated managed forest (plot 2), on wood of fallen *Piceaabies* (decay stage 2), 60.3544°N, 25.0322°E, 2013, Kantelinen 2729 (DNA A466), H.

#### ﻿ Micarea melaeniza

Taxon classificationFungiLecanoralesPilocarpaceae

Hedl.

89697399-C690-5CCD-A8BA-B77F0CA5B732

MycoBank No: 368074

Fig. 3C, D

##### Type.

Bih. Kongl. Svenska Vetensk.Akad. Hand. III, 18: 96 (1892). Type: Sweden, Helsinglandiæ [= Hälsingland], Jerfsö [= Järvsö], VIII 1891. J. T. Hedlund (S L1471! – lectotype, designated by Coppins 1983 [ICN Art. 9.10], further specified here [ICN Art. 9.17], S L1472!, UPS L-005556!, UPS L-171894!, LD 1056591!, isolectotypes).

##### Description.

***Thallus*** endoxylic. Photobiont micareoid, 4–7 µm.

***Apothecia*** absent to numerous (mostly rare), immarginate, subglobose, often becoming tuberculate, black, matt, 0.1–0.3 mm in diam. Hymenium (25–)28–42 µm tall, hyaline or tinged aeruginose green, olive, or rarely purplish brown, often with darker vertical streaks. Epihymenium irregularly pigmented aeruginose green, or rarely sordid brown, sometimes with a purplish tinge. Epihymenium and hymenium K+ olive green, HNO_3_ purple (Cinereorufa-green), or rarely K–. Asci clavate, 22–35 × 10–12 µm. Ascospores ellipsoid to usually ovoid, simple, 5–9 × 2.5–4.0 µm. Paraphyses numerous, of two types: 1.) evenly distributed, branched, thin, c. 0.7–1 µm wide, 2.) scattered or in small fascicles, stout, c. 2 µm wide, coated by greenish pigment. Hypothecium c. 60–120 µm tall, dark brown (Superba-brown), sometimes greenish or with a reddish tinge, often K+ olive green in the upper part (reaction in the greenish Cinereorufa-green pigment), hyphae coated with dark brown pigment. Excipulum not evident.

***Mesopycnidia*** always present, usually numerous, black, sessile or more usually stalked and then 80–300 µm tall, 40–70 µm in diam., stalks simple or branched from the base bearing up to four pycnidia, upper part of the walls greenish-brown and lower part reddish-brown, K+ dull green (the greenish pigment) or sometimes K–. Mesoconidia ellipsoid to short cylindrical 2.5–3.5 × 1.2–1.8 µm. Micro- or macropycnidia not seen.

***Crystalline granules*** not present in apothecia, pycnidia or thallus.

***Chemistry*** no substances detected by TLC (information based on Coppins 1983 and Czarnota 2007).

***Typification*.** In his original description of *Micareamelaeniza*, Hedlund (1892) cited material that he had collected in Järvsö in Hälsingland, but without giving further specimen data. There are five specimens of *M.melaeniza* in S, LD and UPS collected by Hedlund in Järvsö in August 1891 and which all are likely to be part of the original material. Coppins (1983) cited a ‘holotype’ in S, which constitutes a lectotypification following ICN Art. 9.10. There is, however, an additional specimen in S (S L1472) with the same label data, and as Coppins (1983) did not indicate which of these specimens he considered to be the holotype, the lectotypification effectively concerns both specimens. We therefore further specify this by here designating the specimen S L1471 as the lectotype. This specimen was likely the one referred to as holotype by Coppins, as annotation slips from him are included in the envelope. It should be noted that all five type specimens of *M.melaeniza* are homogeneous.

##### Habitat and distribution.

*M.melaeniza* occurs on lignum of conifer stumps and logs. Based on sequenced specimens and type, the species is currently known from the Czech Republic, Finland, Sweden, Ukraine and the Russian Caucasus. In addition, *M.melaeniza* has been reported from Alaska (Spribille et al. 2020), Austria (Berger and Türk 1991, this study) and Mongolia (Palka and Śliwa 2006). Further, it might have been reported as *M.nigella* and could be found after revising specimens.

##### Notes.

In his monograph of European *Micarea* species, Coppins (1983) accepted *M.melaeniza*, and in his interpretation, the species is characterized by having a hymenium with green pigmentation, a dark brown hypothecium without any reaction with K, and black stalked pycnidia containing comparatively short conidia. In the same work, the new species *M.nigella* was described, which should differ from *M.melaeniza* by having a purplish brown, K+ green pigment in the hymenium, hypothecium and pycnidial tissues, and slightly larger mesoconidia (*M.melaeniza*: 2.3–3.6 × 1–1.3 µm vs. *M.nigella*: 3.4–4.3 × 1.2–1.6 µm; Coppins 1983). Czarnota (2007) noted that the amount of purple, K+ green pigment varied considerably in Polish collections determined as *M.nigella*, and suggested that *M.melaeniza* and *M.nigella* could be conspecific. He further noted that Hedlund’s original description could be interpreted as indicating the presence of another pigment, the purple, K+ purple pigment and suggested that the differences between Hedlund’s and Coppins’ descriptions could be due to the studied material having aged (Czarnota 2007).

In our interpretation, *M.melaeniza* is a species with mostly two pigments: (i) a blackish-green, usually K+ green intensifying pigment, mostly located to the epihymenium but sometimes also in the hymenium and the upper part of the hypothecium (Cinereorufa-green) and (ii) a dark brown, K– pigment in the hypothecium (possibly Superba-brown). The description in Coppins (1983) fits our interpretation quite well, except that the specimens have a K+ greenish reaction due to Cinereorufa-green pigment that is not mentioned by Coppins (l.c.) but is, on the other hand, mentioned in the original description of the species by Hedlund (1892) and seen by us in the type specimen. Pigmentation of *M.melaeniza* may be more complex, however. One specimen from the Czech Republic (ZP32013) shows patchily purplish, K+ dark green pigment that might be the Melaena-red pigment (in apothecia concentrated mainly in hymenium as darker streaks). This specimen was originally downloaded to GenBank as *M.nigella*, but it is monophyletic with *M.melaeniza* in our phylogenetic analyses. More sequenced specimens are needed to understand better the pigment profile of *M.melaeniza*.

We considered the possibility of *M.melaeniza* and *M.nigella* being synonymous. A careful study of the type specimens showed morphological differences, e.g. in the size of conidia and pigmentation of apothecia and pycnidia. A brown or purple-brown, K+ green pigment in the hymenium, hypothecium and pycnidia walls (Melaena-red) of *M.nigella* is an important difference between *M.melaeniza* and *M.nigella*, although this is not true for all the studied specimens as was mentioned above. The difference in pigmentation is also visible in nitric acid, i.e. in *M.melaeniza* the hypothecium is mostly HNO_3_– (rarely HNO_3_ intensifying red), and in *M.nigella* HNO_3_+ purple-red. Compared to *M.melaeniza*, *M.nigella* also has longer conidia (3.4–4.5 × 1.2–1.6 µm), slightly shorter hymenium (up to 30 µm) and wider paraphyses (up to 3 µm), as also shown by Coppins (1983).

The molecular study supports the distinction of *M.melaeniza* and *M.nigella* (Fig. 2). Our sequenced specimens form two monophyletic clades, and these specimens are morphologically similar with the type specimens (except for ZP32013 discussed above).

In external appearance, *M.melaeniza* also resembles *M.botryoides*, *M.eurasiatica*, *M.misella* and *M.osloensis*. *Micareabotryoides* is usually not lignicolous, has longer ascospores (8–13(–16) × 2.3–3.7(–4) µm) that are often septate, and longer mesoconidia (Coppins 1983). *Micareaeurasiatica* has similar pigmentation like *M.melaeniza*, but the shape of apothecia is different (adnate vs subglobose), its pycnidia are sessile and mesoconidia are longer (up to 6 µm). *Micareamisella*, on the other hand, can be microscopically distinguished from *M.melaeniza* by the olivaceous pigment that reacts violet instead of dull green in K, and by its hyaline hypothecium (Coppins 1983). *Micareaosloensis* is similar to *M.melaeniza* in many characters, and is a close relative based on our phylogenetic study. However, *M.osloensis* has shorter pycnidia (max. 180 µm), longer mesoconidia 3.5–4.5(–5) × 1.2–1.5 (–1.8) µm, and apothecia and pycnidia are mostly K– (although a higher concentration of Cinereorufa-green pigment is known to occur in some of the C-European specimens).

##### Additional specimens studied.

**Austria**, Niederösterreich, Ybbstaler Alpen, Wildnisgebiet Dürrenstein, Lunz am See Rothwald, Kleiner Urwald, primeval beech dominated forest on a crest above the valley of Moderbach brook, 47°46'31.0"N, 15°06'10.5"E, 1010 m, on wood of snag of *Piceaabies*, 2022, Malíček 16229, Berger, Palice & Vondrák, hb Malíček.

**Czech Republic**, S Bohemia, Šumava Mts, Volary, České Žleby: Radvanovický hřbet - E foothill, managed spruce forest with left old beeches on E-NE-facing slope, 48°54'02"N, 13°48'28"E, on decaying wood of (?)*Picea* stump, 820 m, 2021, Palice 32013, PRA (in GenBank as *M.nigella*: OQ646318); ibid., Horní Vltavice, Zátoň: Jilmová skála Nature Monument, scree old-growth forest (150–200 years old) with maple, beech, sycamore, silver fir etc., 48°57'13"N, 13°47'48"E, 1000–1030 m, on decaying stump, 2014, Malíček 7322, hb Malíček.

**Finland**, Uusimaa, Vantaa, Herukkapuro nature reserve (plot 1), old-growth forest, on wood of a dead stump of *Piceaabies* (decay stage 5), WGS84 60.3215°N, 24.7658°E, 2013, Kantelinen 2430 (DNA A772), H.

**Ukraine**, Ukrainian Carpathians, Nadvirna, Bystrytsia, in valley of stream Dzhurbzinets, c. 3 km south of village Maksymets, 48°28'30"N, 24°18'23"E, 1005 m, on soft wood of coniferous (*Picea*?) stump, 2019, Vondrák 21921, PRA.

**Russia**, Russian Western Caucasus, Adler, Krasnaya Polyana, primeval fir-beech forest below timberline, 43°41'50"N, 40°21'25"E, 1690 m, on soft rotten wood of *Abies* snag, 2019, Vondrák 23358, PRA.

**Sweden**, Ångermanland, Långsele par., VII. 1891. Hedlund, UPS (L-171893).

#### ﻿ Micarea nigella

Taxon classificationFungiLecanoralesPilocarpaceae

Coppins

B0CDBF4B-8047-5259-8166-E989AA6812D4

Fig. 3E, F, G

Micarea nigella Coppins. Bull. Brit. Mus. Nat. Hist. 11(2): 163 (1983). Type: Denmark, Jylland, c. 16 km N of Hobro, Rold Skov, Torstedlund Skov, on conifer stump, lignum, 1979, Coppins 4429 (RBGE! – holotype).

##### Description.

***Thallus*** endoxylic or thin green-grey layer on top of substrate. Photobiont micareoid, 4–7 µm.

***Apothecia*** absent to numerous (mostly rare), immarginate, subglobose, often becoming tuberculate, black, matt, 0.1–0.3 mm in diam. Hymenium 25–30 µm tall, hyaline or tinged dull brown or purplish brown, K+ sordid green, HNO_3_+ purple-red (Melaena-red), often with darker vertical streaks. Epihymenium irregularly pigmented brown to purplish-brown (Melaena-red), sometimes dark greenish, K+ olive green, HNO_3_+ purple (Cinereorufa-green). Asci clavate, 22–30 × 10–12 µm. Ascospores ellipsoid to usually ovoid, simple, 6.5–12 × 2.5–4.0 µm. Paraphyses of two types: 1.) evenly distributed, branched, thin, c. 0.7–1 µm wide, 2.) scattered or in small fascicles, stout, 2–3 µm wide, coated by dark pigment. Hypothecium c. 70–120(–160) µm tall, dark brown with variable amount of purplish tone, K+ olive green, HNO_3_+ purple-red (Melaena-red), hyphae coated with a dark brown pigment. Excipulum not evident.

***Mesopycnidia*** always present, usually numerous, black, sessile or more usually stalked and then 80–300 µm tall, 40–80 µm in diam., stalks simple or branched from the base bearing up to four pycnidia, walls brownish black to purplish black, sometimes olivaceous from the top, K+ dull green and HNO_3_+ purple-red especially in the brown parts (Melaena-red). Mesoconidia ellipsoid or short cylindrical 3.5–4.5(–5) × 1.2–1.8 µm. Micro- or macropycnidia not seen.

***Crystalline granules*** not present in apothecia, pycnidia or thallus.

***Chemistry*** no substances detected by TLC (information based on Coppins 1983 and Czarnota 2007).

##### Habitat and distribution.

*Micareanigella* occurs mainly on lignum of conifer stumps or fallen trunks, sometimes spreading from wood to dead bryophytes. Based on sequenced specimens and the type material, the species is known from the Czech Republic, Denmark (holotype), Great Britain (paratypes), Finland and Sweden. In addition, *M.nigella* has previously been reported from boreal and temperate forests in north-western, central and eastern Europe (e.g. Czarnota 2007).

##### Notes.

In external appearance, *M.nigella* resembles *M.melaeniza*. The differences between these two species are discussed in detail under *M.melaeniza* and Coppins (1983). The species also resembles *M.botryoides*, *M.misella* and *M.osloensis*. *Micareabotryoides* is usually not lignicolous and prefers rain-sheltered microhabitats on various substrata, it has slightly taller pycnidia (up to 400 µm) and longer ascospores (8–13(–16) × 2.3–4 µm) that are often septate (e.g. Coppins 1983). Microscopically, *M.misella* can be distinguished by the olivaceous pigment that reacts violet instead of dull green in K, and by its hyaline hypothecium (Coppins 1983; Czarnota 2007). *Micareaosloensis*, on the other hand, is usually K– and its pycnidia are shorter. However, our study includes specimens that are difficult to identify by morphological characters, especially between *M.melaeniza*, *M.nigella* and *M.osloensis*.

One of the distinguishing characteristics of *M.nigella* is the Melaena-red pigment (K+ green, HNO_3_+ purple-red) in the hymenium, hypothecium and pycnidia. In the literature, the pigment is described as `purple` (Coppins 1983; Meyer and Printzen 2000; Czarnota 2007). However, based on our study, the pigment is mostly brown, sometimes with a purplish tinge. The holotype of *M.nigella* has the Melaena-red pigment, that looks brown with a purplish tinge, but of our three sequenced specimens (Fig. 2, Table 1), one (collection Kantelinen 1971) has no purplish tone, whereas the other two collections (Kantelinen 1974, 1921) have easily detected amounts of purple. Interpreting the colouration can be difficult and confusing, but maybe a helpful hint is that the pigment is always K+ green, even if it looks brown in water. According to our study, the K+ green reaction mostly disappears in 30 minutes.

Occasionally, *M.nigella* also has a third pigment, the Melaenida-red (K+ purple). This pigment was not found in the Finnish specimens but is sometimes seen in the Central European specimens included in this study and mentioned also by Coppins (1983) and Czarnota (2007). The Melaena-red and Melaenida-red pigments can be intermixed and appear in varying concentrations.

##### Additional specimens studied.

**Czech Republic, Central Bohemia**, Brdy Protected Landscape Area, Míšov, Na Skalách Nature Reserve, old-growth beech forest, small scree with rock outcrops and sparse spruce forest in NW part of the protected area, 49°36'20"N, 13°45'56"E, 715–740 m, on stump, 2023, Malíček 16287, hb Malíček. **Western Bohemia**, Domažlice, Český les Protected Landscape Area, Pec, Bystřice Nature Reserve, natural mixed forest up to 150 years old, 49°22'56"N, 12°48'39"E, 650–750 m, on lying decaying trunk, 2015, Malíček 8029 (hb Malíček); ibid., Tachov Lesná: managed spruce forest 1 km NE of Knížecí strom Hill (829 m), 49°46'13.0"N, 12°29'28.3"E, 750 m, on stump of *Piceaabies*, 2019, Malíček 13161 & Rydlo, hb Malíček. **Southern Bohemia**, Prachatice, Šumava National Park, Nová Pec old-growth beech-spruce forest on N-facing slope of Mt Hraničník (1281 m), 48°45'13"N, 13°54'17"E, 1170 m, on decaying wood, 2017, Malíček 11296, hb Malíček. **Eastern Bohemia**, Žďár nad Sázavou, Žďárské vrchy Protected Landscape Area, Svratka managed beech-spruce forest on NW-facing slope of Bubnovaný kopec Hill (780 m), 49°42'41.6"N, 16°05'13.7"E, 775 m, on stump of *Piceaabies*, 2020, Malíček 13865 & Sejfová, hb Malíček. Svratka forest mosaic 0.2 km SSE of Spálený kopec Hill (766 m), 49°43'28.0"N, 16°06'23.3"E, 755 m, on stump of *Piceaabies*, 2020, Malíček 13868 & Sejfová, hb Malíček. Svitavy, Česká Třebová, Psí kuchyně Nature Reserve, old-growth beech forest 0.1 km NW of Psí kuchyně Hill (526 m), 49°50'38.3"N, 16°26'47.5"E, 505 m, on lying wood of *Fagussylvatica*, 2020, Malíček 13975 & Rydlo, hb Malíček. **Silesia**, Beskydy Protected Landscape Area, Horní Lomná, Velký Polom Nature Reserve, valley of a brook with old-growth, beech predominated forest in the E part of the protected area, 49°30'36"N, 18°41'00"E, 860–910 m, on stump of *Piceaabies*, 2021, Malíček 14664 & Sejfová, hb. Malíček; ibid., gorge on NW-facing slope of Mt Velký polom (1067 m), c. 49°30'25"N, 18°40'04"E, 950–1000 m, on stump, 2021, Malíček 14649, Hlisnikovský & Sejfová, hb Malíček; ibid., Karolinka, Malý Javorník Nature Reserve, old-growth spruce-beech forest, 49°18'20.9"N, 18°17'18.5"E, 880–970 m, on wood of stump, 2023, Malíček 16392, hb Malíček; ibid., Valašská Bystřice, Kutaný Nature Reserve, old-growth beech-silver fir forest, 49°22'15.6"N, 18°6'0.4"E, 610–770 m, on fallen wood, 2020, Malíček 14246 & Konečná, hb Malíček. Jeseníky Protected Landscape Area, Karlova Studánka, old-growth spruce forest on SE-facing slope in valley of Bílá Opava Brook, along tourist path 1.1 km NE of Ovčárna, 50°04'37"N, 17°15'06"E, 1170–1200 m, on roots of *Piceaabies*, 2015, Malíček 8547, hb. Malíček.

**Finland, Pohjois-Karjala**, Lieksa, Koli National Park (plot 9), East side, old-growth forest, on wood of fallen *Piceaabies* (decay stage 2), 63.1033°N, 29.8140°E, 2013, Kantelinen 1971 (DNA A589), H; ibid., Kantelinen 1974 (DNA A588), H; ibid., Kantelinen 1921 (DNA A572), H. **Etelä-Häme**, Hämeenlinna, Evo (plot 8), protected old managed forest, on wood of a *Piceaabies* stump (decay stage 5), 61.2088°N, 25.1363°E, 2013, Kantelinen 2851 (DNA A769), H; ibid., Kantelinen 2881 (decay stage 4, DNA A764), H.

**Slovakia**, Eastern Slovakia, Bukovské vrchy Mts., Nová Sedlica, protected area Stužica, NNE-facing slope of Temný vršok Mt. (838 m), old-growth beech forest, 49°04'11"N, 22°32'26"E, 750–820 m, on stump of *Abiesalba*, 2013, Malíček 6511 & Vondrák, hb Malíček.

#### ﻿ Micarea osloensis

Taxon classificationFungiLecanoralesPilocarpaceae

(Th. Fr.) Hedl.

F553C3E8-6516-59CD-88A7-9A028398A035

MycoBank No: 627666

Fig. 4A, E

##### Type.

Bih. Kongl. Svenska Vetensk.Akad. Hand. III, 18: 97 (1892). *Lecideaosloensis* Th. Fr. Lich. Scand. 2: 524 (1874). Type: Norway, Oslo, In cacumine Ryenbjerget, 10 July 1874. N.G. Moe (UPS L-153276! – holotype).

**Figure 4.**
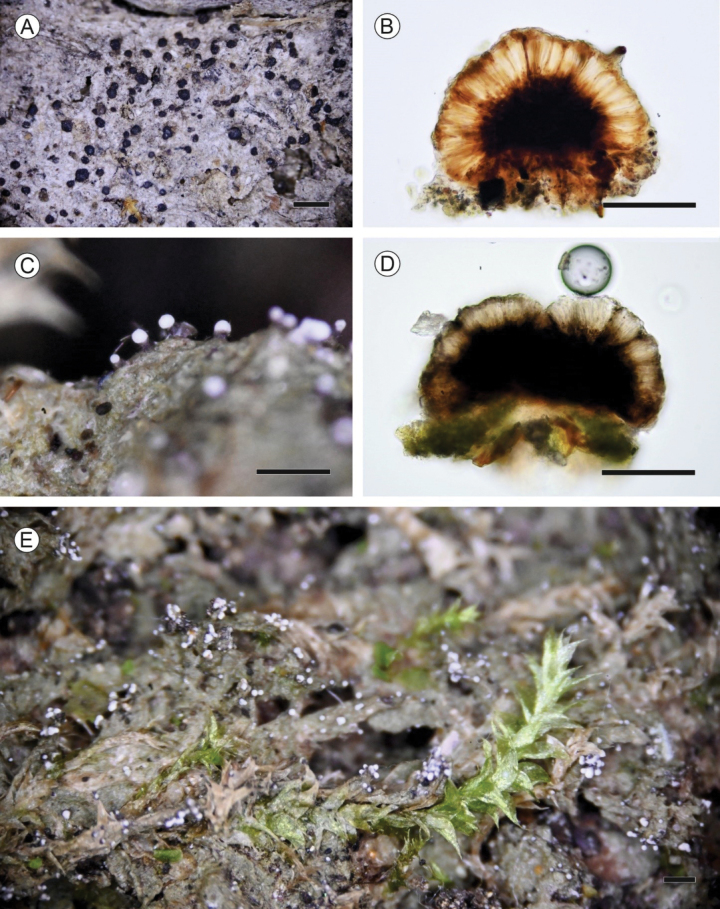
Morphological and anatomical features of old and new *Micareaosloensis* collections **A, B** old *Micareaosloensis***A** habit, apothecia on soil (Holotype) **B** apothecial section in water (Palice 11684, H) **C–E** new *Micareaosloensis* (Kantelinen 2648, H) **C** mesopycnidia extruding mesoconidia as a white drop **D** apothecial section in water **E** mesopycnidia on dead wood and mosses. Scale bars: Habit 0.5 mm (**A, C, E**); Apothecial sections 100 µm (**B, D**).

##### Description.

***Thallus*** endoxylic or visible as a thin pale greenish-grey to dark green-grey layer on top of substrate. Photobiont micareoid, 4–7 µm in diam.

***Apothecia*** infrequent or rare, absent or numerous, immarginate, convex to hemispherical, dark brown to black, matt, simple, 0.1–0.2(–0.3) mm in diam. ***Hymenium*** 30–50 µm tall (Coppins c. 30 µm tall), hyaline, sometimes olivaceaous, often with warm brown vertical streaks, K–, HNO_3_– (Superba-brown). ***Epihymenium*** warm dark brown to blackish (Coppins: red-brown), rarely greenish, mostly K– but sometimes K+ green, HNO_3_– or HNO_3_+ purplish (Cinereorufa-green). ***Paraphyses*** of two type: 1.) hyaline or coated with a brown pigment, thick, 2–3 µm wide, simple or branched, sometimes wider from apices, often uneven in shape, abundant, sometimes concentrated into fascicles, 2.) thinner, c. 1–1.5 µm wide, rarely branched, rare. ***Asci*** cylindrical to cylindrical-clavate, 30–40 × 10–12 µm (Coppins: 26–30 × 11–13 µm). ***Ascospores*** 7–10 × (2.5–)3.0–3.5 (–4.0) µm (Coppins: 6–9.5 × 3–4 µm), ellipsoid, cylindrical or sometimes roughly shaped, 0(–1) sept. ***Hypothecium*** warm dark brown (Coppins: red-brown), composed of hyaline hyphae 1–2 µm wide surrounded by brown pigment giving it an unevenly coloured/randomly spotted appearance, K–, HNO_3_– (Superba-brown), c. 85 µm tall.

***Mesopycnidia*** often numerous and crowded, sometimes absent, simple or branched from the base, emergent or shortly stalked ca. 50–180 µm tall, 40–70 µm in diam., dark brown to blackish, walls brown, greenish brown from the top with a warm brown lower part, K– (Superba-brown) or sometimes K+ green, HNO_3_– or HNO_3_+ purplish (Cinereorufa-green), usually extruding white mass of conidia that may merge with neighbouring conidia. Mesoconidia ellipsoid or short cylindrical 3.5–4.5(–5) × 1.2–1.5 (–1.8) µm. Micro- or macropycnidia not seen.

***Chemistry*** no substances detected by TLC (Coppins 1983).

***Crystalline granules*** not present in apothecia, pycnidia or thallus.

##### Habitat and distribution.

The type of *M.osloensis* occurs on soil. Another morphologically identical specimen collected in 2007 occurs on bark of decaying trunk (Palice 11684). Our newer specimens occur on bark, dead wood and dead mosses. The type specimen was collected from Norway from a woodland clearing on the site of an old bonfire, and the newer specimens are from the Czech Republic, Finland, Sweden and Ukraine. In the Czech Republic, *M.osloensis* occurs commonly from middle to montane elevations. It appears to be toxitolerant and is known in areas with higher levels of air pollution in the past (i.e. acidification by acid rain). The typical habitats are bark on bases and roots of *Fagussylvatica*, *Larixdecidua*, *Piceaabies*, *Pinussylvestris*. It is abundant also on dead wood and dead bark on stumps, fallen trunks and snags. In Finland, *M.osloensis* is likely relatively common but overlooked in coniferous forests on bark, dead wood and dead mosses. In both countries, *M.osloensis* is known from managed and old-growth forests.

##### Notes.

The two previously known *M.osloensis* specimens, including the type, have not been sequenced, although an unsuccessful sequencing attempt of a specimen collected by Palice (11684) was made by Kantelinen in 2011, and therefore we cannot compare our new specimens to the type of *M.osloensis* using DNA. Subtle morphological features differentiate the type from new specimens, i.e. taller hymenium and asci. Most of the new specimens are K– and have only the Superba-brown pigment, similar to the type. However, some specimens have a K+ greenish, HNO_3_+ purple reaction in the epihymenium and pycnidial walls suggesting the presence of the Cinereorufa-green pigment which is not known from the type of *M.osloensis*. Specimens with the Cinereorufa-green pigmentation appear to be more frequent in the Czech Republic. The Finnish specimens have sometimes olivaceous tones that are K– but slightly HNO_3_+ purple.

Another difference between the type of *M.osloensis* and our newly sequenced specimens is reproduction. The type specimen has apothecia and no pycnidia. The new specimens, on the other hand, often have shortly stipitate pycnidia. Our specimens appear to be dimorphic, however, so that the specimens represent either sexual (rare) or asexual reproduction modes which are monophyletic in DNA level.

Because of overlapping variation in reproduction and pigmentation between the type of *M.osloensis* and our new specimens, we cannot exclude the possibility that they are conspecific. On the other hand, we also cannot exclude the possibility that the new specimens represent a yet undescribed taxon in the *M.melaeniza* group.

*M.osloensis* resembles *M.eurasiatica*, *M.melaeniza*, *M.misella* and *M.nigella*. The most important characters of *M.osloensis* are the combination of sessile to shortly stalked pycnidia, mesoconidia of the size 3.5–4.5(–5) × 1.2–1.5 (–1.8) µm, warm-brown, sometimes olivaceous colouration in apothecia and pycnidia (Coppins: red-brown), and often a K– reaction. *Micareaeurasiatica* sp. nov. has mostly sessile to emergent pycnidia and bigger mesoconidia ((4–)4.5–6.0 × 1.2–1.5(–1.8) µm). *Micareamisella*, on the other hand, has a K+ violet reaction in the epihymenium and pycnidia (Coppins 1983; Czarnota 2007). *Micareanigella* has similar mesoconidia and spore size, but its pycnidia are usually taller and it has the brown/purple-brown Melaena-red pigment (K+ green) in hypothecium and pycnidia.

Based on our phylogenetic analyses, *M.osloensis* and *M.melaeniza* are sister species. They have morphological similarities including pigmentation and spore size. However, *M.osloensis* has slightly larger mesoconidia, shorter pycnidia and wider often roughly shaped paraphyses. The concentration of the Cinereorufa-green pigment (K+ green, HNO_3_+ purple) appears to vary in both taxa, but especially in *M.osloensis*. *Micareamelaeniza* is mostly K+ green in epihymenium, hymenium, upper hypothecium and pycnidia. *Micareaosloensis*, on the other hand, is rarely K+ green and then from the epihymenium and pycnidia.

##### Additional specimens studied.

**Czech Republic**, **Northern Bohemia**, Jizerské hory Mts, Josefův Důl: valley of Jedlový potok, protected zone of nature reserve Jedlový důl, fragment of fir-beech old-growth forest, 50°47'45"N, 15°14'47.5"E, on dry wood of old conifer stump, 780 m, 2020, Palice 30266, PRA (in GenBank as *M.nigella*: OQ646320); ibid., Hejnice, Jizerskohorské bučiny National Nature Reserve, valley of Velký Štolpich brook, S of Ořešník Mt. (800 m), ca 50°51'13"N, 15°11'13"E, 660 m, on base of *Fagussylvatica*, 2013, Malíček 6020, hb Malíček. Lužické hory Protected Landscape Area, Mařenice, Horní Světlá: managed spruce-beech forest on E-facing slope, 0.3 km NNW of Kopřivnice Hill (638 m), 50°50'13.6"N, 14°37'56.6"E, 600 m, at base of *Piceaabies*, on stump of *Picea*, 2020, Malíček 14019, 14020 & Rydlo, hb Malíček. **Central Bohemia**, Brdy Protected Landscape Area, Míšov, Na Skalách Nature Reserve, old-growth beech forest, small scree with rock outcrops and sparse spruce forest in NW part of the protected area, 49°36'20"N, 13°45'56"E, 715–740 m, at base of *Fagussylvatica*, 2023, Malíček 16281, hb Malíček. Brdy Hills, Rožmitál pod Třemšínem, Nepomuk: managed forest 0.4 km N of Praha Hill (862 m), 49°39'48"N, 13°49'06"E, 825 m, at base of *Larixdecidua*, 2018, Malíček 12007 & Vondrák, hb Malíček; ibid., Strašice, managed mixed forest 3 km E of village, 49°43'34"N, 13°47'56"E, 610 m, at base of *Larixdecidua*, 2018, Malíček 12000 & Vondrák, hb. Malíček. Příbram, Brdy Hills, Jince managed coniferous forest 0.5 km SSE of Velcí pond, 49°45'17"N, 13°56'34"E, 600 m, at base of *Larixdecidua*, 2018, Malíček 12016 & Vondrák, hb Malíček. **Eastern Bohemia**, Rychnov n. Kněžnou, Orlické hory Protect. Lands. Area, Rokytnice v Orlických horách, Černý důl Nature Reserve, fragment of old-growth beech-spruce-silver fir forest, along brook, 50°12'01.4"N, 16°31'18.2"E, 800–810 m, on bark of stump of *Piceaabies*, 2012, Malíček 4536 et al., hb Malíček. Žďár nad Sázavou, Žďárské vrchy Protected Landscape Area, Svratka fragment of old beech predominated forest 0.6 km SW of Spálený kopec Hill (766 m), 49°43'18.2"N, 16°06'11.4"E, 750 m, on stump of *Piceaabies*, 2020, Malíček 13864 & Sejfová, hb Malíček; ibid., Svratka, Pustá Rybná: spruce-beech forest on S-facing slope of Kaštánkův kopec (753 m), 49°43'03.9"N, 16°06'46.4"E, 740 m, on stump of *Piceaabies*, 2020, Malíček 13953, hb Malíček. Svitavy, Česká Třebová managed spruce forest 3 km W of Opatov, 49°49'47.6"N, 16°27'40.0"E, 445 m, on stump of *Piceaabies*, 2020, Malíček 13970 & Šámalová, hb Malíček. **Western Bohemia**, Český les Protected Landscape Area, Tachov, Lesná: young beech forest 0.8 km E of Knížecí strom Hill (829 m), 49°45'54.2"N, 12°29'18.5"E, 780 m, on stump of *Piceaabies*, 2020, Malíček 13784 & Rydlo, hb Malíček; ibid., spruce-beech forest 1 km NE of Knížecí strom Hill (829 m), 49°46'17.3"N, 12°29'18.1"E, 770 m., at base of *Piceaabies*, 2019, Malíček 13167 & Rydlo,hb Malíček; ibid., Pec, Bystřice Nature Reserve, natural mixed forest up to 150 years old, 49°22'56"N, 12°48'39"E, 650 m, at base of *Piceaabies*, 2015, Malíček 8028, hb Malíček. Kdyně, Mezholesy: managed mixed forest 0.3 km SE of Koráb Hill (773 m), 49°23'37.7"N, 13°04'44.1"E, 750 m, at base of *Piceaabies*, 2019, Malíček 13371 & Rydlo, hb Malíček. **Southern Bohemia**, Novohradské hory Mts, Horní Stropnice, NPP Hojná Voda, fragment of primeval forest predominated by beech, 48°42'20"N, 14°45'08"E, 840–870 m, on snag, 15 October 2019, Malíček 13500, herb. Malíček. Šumava Mts., Volary, Nová Pec, 740 m, 48°49'11.2"N, 13°56'12.151"E, on bark of *Pinussylvestris* at base of trunk, 2017, Vondrák 19007, PRA. Prachatice, Šumava Protected Landscape Area, Kubova Huť, Boubínský prales National Nature Reserve, managed spruce forest c. 120 years old, 0.4 km NNE of top of Mt Boubín (1362 m), 48°59'42.2"N, 13°49'05.7"E, 1275 m, on decaying stump 2015, Malíček 8349 & Palice, hb Malíček. Jindřichův Hradec, Javořická vrchovina Hills, Stráž nad Nežárkou, Sedlo: managed coniferous forest SSE of Otínský kopec Hill (538 m), 49°02'44.0"N, 14°59'45.7"E, 530 m, on stump, 2020, Malíček 13817, hb Malíček; ibid., Lásenice: mixed forest S of Šemburský rybník, 49°03'34.6"N, 14°59'55.2"E, 520 m, on stump, 2020, Malíček 13821, hb. Malíček; ibid., managed coniferous forest between Nová Ves and Sedlo, 49°03'36.1"N, 15°00'55.3"E, 560 m, at base of *Pinussylvestris*, 2020, Malíček 13824, hb Malíček. Tábor Chýnov, young beech forest SSE of Blanička, 49°28'00.6"N, 14°50'34.9"E, 690 m, on fallen wood, 2020, Malíček 13834 & Rydlo, hb Malíček; ibid., mixed forest SE of Batkovy Hill (724 m), 49°27'43.3"N, 14°50'00.9"E, 700 m, at base of *Larixdecidua*, 2020, Malíček 13838 & Rydlo, hb Malíček. **Northern Moravia**, Jeseník, Jeseníky Protected Landscape Area, Bělá pod Pradědem, Vysoký vodopád, Nature Reserve, valley of Studený p. brook, ca. 50°06'57"N, 17°12'10"E, 900–1000 m, on base of *Piceaabies*, 2012, Malíček 5102, hb Malíček. Šumperk, Králický Sněžník Mts, Staré Město: Mt. Králický Sněžník, c. 100 years old spruce forest on S-facing slope 0.1 km SE of Františkova chata, 50°12'05.2"N, 16°51'28.3"E, 1210 m, at base of *Piceaabies*, 2015, Malíček 8381, Kocourková & Vondrák, hb Malíček. **Eastern Moravia**, Beskydy Protected Landscape Area, Bílá, Salajka: beech dominated forest 0.6 km SE of gamekeeper’s house, 49°24'37.9"N, 18°25'39.5"E, 730 m, on decaying stump, 2019, Malíček 13344 & Rydlo, hb Malíček; ibid., Frenštát pod Radhoštěm, Kněhyně-Čertův mlýn National Nature Reserve, W-facing slope of Kněhyně Mt. (1257 m), old-growth spruce forest above red-marked tourist path, 49°29'57"N, 18°18'38"E, 1080–1100 m, on stump of *Piceaabies*, 2013, Malíček 6090 & Vondrák, hb Malíček. **Western Moravia**, Žďár nad Sázavou, Žďárské vrchy Protect. Landsc. Area, Cikháj, Žákova hora National Nature Reserve, beech virgin forest, 49°39'18"N, 15°59'35"E, 750–800 m, on stump of *Fagus*, 2012, Malíček 5110 & Syrovátková, hb Malíček. **Silesia**, Bruntál, Jeseníky Protected Landscape Area, Karlova Studánka, managed spruce forest (c. 100 years old) on N-facing slope in valley of Bílá Opava Brook, 1.8 km ENE of Ovčárna, 50°04'35"N, 17°15'53"E, 1170–1180 m, on bark of *Piceaabies*, 2015, Malíček 8490, Kocourková, Vondrák & Zemanová, hb Malíček; ibid., Praděd National Nature Reserve, old-growth spruce forest c. 200 years old on E-facing slope of Mt Vysoká hole (1464 m), 0.2 km WNW of Eustaška hut, 50°03'35"N, 17°15'12"E, 1220 m, at base of *Piceaabies*, 2015, Malíček 8563, Kocourková, Palice & Vondrák, hb Malíček. Frýdek-Místek, Beskydy Protected Landscape Area, Ostravice, Mazák National Nature Reserve, old-growth spruce forest with intermixed sycamores on W-facing slope of Mt Lysá hora (1323 m), 49°32'41"N, 18°26'43"E, 1200 m, on bark of *Piceaabies*, 2016, Malíček 9743 & Palice, hb Malíček.

**Finland, Pohjois-Karjala**, Lieksa, Koli National Park (plot 9), East side, old-growth forest, on bark of fallen *Piceaabies* (decay stage 3), 63.1033°N, 29.8140°E, 2013, Kantelinen 1865 (DNA A575, apothecia and pycnidia), H. Ibid., on wood of fallen *Piceaabies* (decay stage 4), 2013, Kantelinen 1909 (DNA A583), H. Ibid., on wood of dead standing *Piceaabies* (decay stage 3), Kantelinen 1685 (DNA A574), H. Ibid., on wood of fallen *Piceaabies* (decay stage 5), 2013, Kantelinen 2003 (DNA A594), H. Ibid., on wood of fallen *Piceaabies* (decay stage 3), 2013, Kantelinen 1923 (DNA A573), H. **Etelä-Häme**, Hämeenlinna, Evo (plot 8), protected old managed forest, on wood/mosses of a *Piceaabies* stump (decay stage 5), 61.2088°N, 25.1363°E, 2013, Kantelinen 2899 (DNA A736). Ibid., on wood of *Piceaabies*, 2013, Kantelinen 1686 (DNA A768), H. Ibid., Rajakallio, boreal forest on a bouldery slope, forest of *Picea*, *Pinus* and *Betula*, 61°15.27'N, 025°06.43'E, on bark of rotten wood among boulders, 2007, Palice 11684, conf. Coppins, H. **Uusimaa**, Tuusula, west of Korso, shaded and dense *Piceaabies* dominated managed forest (plot 2), on wood of a *Piceaabies* stump (decay stage 5), 60.3544°N, 25.0322°E, 2013, Kantelinen 2643 (DNA A484), H.

**Sweden, Jämtland**, Kall par., about 850 m NW of the northwestern tip of Lake Spjuttjärnen, S side of stream Konäsån, on stump of *Betulapubescens* in old-growth *Piceaabies* forest, 63°34'30"N, 13°04'05"E, elev. 440 m, 2022, Svensson 4335, UPS L-1091180.

**Ukraine.** Eastern Carpathians, Nadvirna, Bysrytsia, N of hill Skali verkhni, 48°27'48.492"N, 24°18'35.46"E, 1233 m, on bark of *Piceaabies*, 2019, Vondrák 22217, PRA.

## ﻿Discussion

Our aim was to clarify systematics and species boundaries among *Micareamelaeniza* and similar-looking species. We propose the new species *Micareaeurasiatica*, characterized by the combination of cylindrically shaped, sessile to emergent pycnidia with greenish-black walls, long mesoconidia (up to 6 µm in length), a K+ olive green reaction and by mostly occurring in the anamorphic stage. The species is known from Japan and Finland. We also discovered another, putatively new species, marked *Micarea* sp. in the phylogeny that we refrain from describing because of insufficient morphological data and few available collections. This putative new species has sessile to emergent, brownish black pycnidia with a purple tinge and a K+ green reaction, but apothecia are few (see Table 2). Despite our efforts, we did not find or sequence *M.olivacea*, a species that could be related to the *M.melaeniza* group.

Generally, the species in the *M.melaeniza* group are challenging to identify because they are small, have relatively few morphological characters and because the current literature is not up to date particularly in relation to pigmentation (Coppins 1983; Czarnota 2007). The challenges while identifying our fresh specimens underline this issue, and are discussed in the notes under *M.melaeniza*, *M.nigella* and *M.osloensis*. According to our study, the species in the *M.melaeniza* group are characterized by having a thin or endosubstratal thallus and dark, sessile to stipitate mesopycnidia that often extrude a white mass of conidia at their top. Apothecia are often absent, but when present, they are 0.1–0.3 mm wide and brown to black in colour. They have hyaline or slightly coloured hymenium, dimorphic paraphyses, simple spores and dark hypothecium where hyphae are surrounded by brown pigment giving them an unevenly coloured appearance, also noted by Coppins (1983). Based on results of this study, the size of pycnidia and mesoconidia, and to some extent also the general pigmentation in K and HNO_3_ are the most useful morphological characters in separating the species, however in some cases DNA sequencing is the only reliable way for identification. *Micareaeurasiatica*, and *M.olivacea* develop sessile to emergent pycnidia, *M.osloensis* emergent to shortly stipitate pycnidia, and *M.melaeniza* and *M.nigella* develop stipitate pycnidia. *Micareademinuta* does not develop distinctive stalked pycnidia, at least according to current knowledge. The Cinereorufa-green pigment (K+ green, HNO_3_+ purple) is present in pycnidia and apothecia of *M.eurasiatica*, *M.deminuta*, *M.melaeniza*, *M.nigella*, *M.olivacea* (according to Coppins 1983) and occasionally in *M.osloensis* in varying concentration, accompanied by the Superba-brown pigment (K–). The brown or purple-brown Melaena-red pigment (K+ dull sordid green) is present in *M.nigella* (and possibly in one specimen of *M.melaeniza*) and is sometimes intermixed with the Melaenida-red pigment (K+ purplish-red) (Table 3).

Our study indicates that the pigmentation of some species may correlate with geography. In the central European specimens some pigments are encountered more often or higher concentrations than in Fennoscandia. For example, in the Fennoscandian specimens of *M.osloensis*, the Cinereorufa-green pigment is barely visible even when using HNO_3_, and the Melaenida-red pigment in *M.nigella* was not found at all. In the central European specimens, however, both pigments were found, and sometimes in relatively high concentrations. The central European material also includes samples that appear to be morphologically “intermediate” between species, for example *M.melaeniza* (ZP32013) may have Melaena-red pigment like *M.nigella*, although its mesoconidia size and DNA profile is similar to *M.melaeniza*. Some of the “intermediate” specimens have not been sequenced and therefore we do not know their identity or whether they are undescribed species. Obviously, more work is needed to understand the pigments and species in this group.

Because of these morphological challenges, we even considered that the species in the *M.melaeniza* group are conspecific, i.e. variation of just one species. However, we excluded this possibility because of several reasons 1) molecular differences, 2) existing morphological differences, even though they may be hard to interpret, 3) the type specimens studied are not morphologically conspecific, 4) the types correspond in morphology to most of our specimens. Based on morphology, one might suggest that *M.melaeniza*, *M.nigella* and *M.osloensis* are variation of one species, but according to our phylogenies, they are not monophyletic without *M.deminuta*, *M.eurasiatica* and *Micarea* sp. Even between *M.melaeniza* and *M.nigella*, the pigmentation is mostly different (Superba brown vs. Melaena-red), and although these pigments may look quite similar to the human eye, they may have different ontologies and evolutionary paths.

According to morphological studies by Coppins (1983), *M.botryoides* and *M.melaeniza* are relatives (group G) and possibly close to *M.contexta*, *M.eximia*, *M.nigella* and *M.olivacea* (group H). Our Bayesian and ML phylogenies conclude that groups G and H are intermixed, however, based on DNA sequencing *M.eximia* is likely not a close relative of the *M.melaeniza* group (unpublished data) and molecular relationships of *M.olivacea* are still unknown. Our phylogenetic analyses also show that *M.melaeniza* and *M.osloensis* are sisters, a relationship that has not been noted in previous publications, but the latter clade is not supported (0.68 / 74). A closer look at the sequences and alignment shows that there are nucleotide differences in the mtSSU region between *M.melaeniza* and *M.osloensis* (ca. 1–2% between specimens Kantelinen 2430 and 1923), but the ITS regions are nearly identical with only two nucleotide differences. At least three conclusions could be drawn from these results. 1) The two clades are, in fact, two species as we suggest in our study. This conclusion is supported by molecular and morphological data, to some extent at least, 2) The two clades are conspecific and represent morphological and molecular variation of *M.melaeniza*, 3) Our new specimens of *M.osloensis* are not conspecific to the type of *M.osloensis* or *M.melaeniza*, but instead a scientifically undescribed species. Any conclusion we make suffers from the uncertainty caused by the *M.melaeniza* and *M.osloensis* type specimens not having sequenced, which means that we cannot compare our sequences to the types and the connections between fresh and old specimens are based solely on morphology. This is also the reason why we refrain from describing our new specimens of *M.osloensis* as a new species to science. The type specimens are over 100 years old and hence likely beyond successful DNA sequencing – based on our experience, sequences are usually difficult to get from *Micarea* specimens just over 3 years old, and nearly impossible when over 6 years old.

All species in the *M.melaeniza* group are either obligate or facultative lignicoles. According to a previous study, the wood-inhabiting lifestyle of *Micarea* species influences their reproductive biology: obligate lignicoles primarily reproduce asexually, likely due to the transient nature of decaying wood, which imposes a time constraint on the species occupying it (Kantelinen et al. 2022a). Asexual reproduction via mesoconidia is likely a more rapid and efficient strategy than sexual reproduction. In the here studied *M.melaeniza* group and relatives, several species are found mostly asexual, viz. *M.melaeniza*, *M.nigella*, *M.osloensis*, *M.eurasiatica*, *M.anterior* and *M.substipitata*.

Based on our field experience as well as previous works by Coppins (1983) and Czarnota (2007), the species in the *M.melaeniza* group are probably common in boreal and hemiboreal forests, both in natural and managed forests. In spite of this, their small size and often anamorphic lifestyle make them easily overlooked, resulting in rare mentions in ecological studies or species inventories. We hope that the morphological and molecular features presented in this study will pave the way for future research endeavors.

## Supplementary Material

XML Treatment for Micarea eurasiatica

XML Treatment for Micarea melaeniza

XML Treatment for Micarea nigella

XML Treatment for Micarea osloensis

## ﻿Additional information

### Author ORCIDs

Annina Kantelinen https://orcid.org/0000-0001-8664-7662

Måns Svensson https://orcid.org/0000-0003-1664-8226

Jiří Malíček https://orcid.org/0000-0002-3119-8967

Jan Vondrakhttps://orcid.org/0000-0001-7568-6711

Göran Thor https://orcid.org/0000-0003-1166-6898

Zdeněk Palice https://orcid.org/0000-0003-4984-8654

Stanislav Svoboda https://orcid.org/0000-0001-9797-4984

Leena Myllys https://orcid.org/0000-0002-9566-9473

### Data availability

All of the data that support the findings of this study are available in the main text.
